# Artificial neural network models for early diagnosis of hepatocellular carcinoma using serum levels of α-fetoprotein, α-fetoprotein-L3, des-γ-carboxy prothrombin, and Golgi protein 73

**DOI:** 10.18632/oncotarget.19298

**Published:** 2017-07-17

**Authors:** Bo Li, Boan Li, Tongsheng Guo, Zhiqiang Sun, Xiaohan Li, Xiaoxi Li, Lin Chen, Jing Zhao, Yuanli Mao

**Affiliations:** ^1^ Center for Clinical Laboratory, 302 Millitary Hospital, Beijing, China; ^2^ Graduate student team, Medical University of PLA, Beijing, China

**Keywords:** hepatocellular carcinoma, artificial neural network, serum tumor biomarker

## Abstract

More than 70% of hepatocellular carcinoma (HCC) cases develop as a consequence of liver cirrhosis (LC). Here we have evaluated the diagnostic potential of four serum biomarkers, and developed models for HCC diagnosis and differentiation from LC patients. Serum levels of α-fetoprotein (AFP), AFP-L3, des-γ-carboxy prothrombin (DCP), and Golgi protein 73 (GP73) were analyzed in 114 advanced HCC patients, 81 early stage HCC patients, and 152 LC patients. Multilayer perceptron (MLP) and radial basis function (RBF) neural networks were used to construct the diagnostic models. Using all stages, HCC diagnostic models had a higher sensitivity (>70%) than the individual serum biomarkers, whereas only early stage HCC diagnostic models had a higher specificity (>80%). The early stage HCC diagnostic models could not be used as HCC screening tools due to their low sensitivity (about 40%). These results suggest that a combination of the two models might be used as a screening tool to distinguish early stage HCC patients from LC patients, thus improving prevention and treatment of HCC.

## INTRODUCTION

Liver cancer is the sixth most common cancer throughout the world, but it is the third leading cause of cancer-related death due to its very poor prognosis. Hepatocellular carcinoma (HCC) is the major histological subtype of liver cancer. The major risk factors of HCC are infections with the hepatitis B and C viruses, which increase the risk of liver cancer by about 20-fold [1]. More than 90% of HCC cases develop as a consequence of underlying liver diseases, and liver cirrhosis (LC) occurs in 80% of HCC cases [2–4]. More than 60% of patients are diagnosed with late-stage disease after metastasis has occurred [5], resulting in an overall 5-year survival rate of < 16% [6]. However, if appropriate treatments are performed in early stages, the 5-year survival rates of HCC patients exceed 75%, highlighting the need to diagnose HCC at early stages in order to achieve the greatest possibility of curative treatment [7]. According to the American Association for the Study of Liver Diseases (AASLD) practice guidelines, curative treatment can be performed in the early stage of HCC (BCLC 0-A), while in the advanced stages (BCLC B-D), only palliative or symptomatic treatments are available [8].

The AASLD guidelines also recommend that α-fetoprotein (AFP) and ultrasound examination be used for HCC surveillance in hepatic cirrhosis population, but early stage HCC can be hardly differentiated from cirrhotic nodules because they have similar features on imaging [9–11]. AFP has been used as an HCC serum biomarker for many years, but its sensitivity is only about 39%-65% [12]. Several other tumor markers have been reported as good complements to AFP and have been used in clinical diagnosis, including lens culinaris agglutinin reactive AFP (AFP-L3), des-γ-carboxy prothrombin (DCP) and Golgi protein 73 (GP73) [13–18], but they do not meet the clinical requirements for early stage HCC diagnosis.

Artificial neural network (ANN) is a mathematical model that simulates the structure of biological neural networks. It possesses the characteristics of parallel information processing, distributed information storage, high non-linearity, good fault-tolerance and strong self-learning, self-organizing, and adaptive ability [19]. ANN has been widely applied in the fields of disease diagnosis and prediction [20–24]. The aim of this study was to develop effective HCC diagnostic models using ANN and four serum tumor biomarkers (AFP, AFP-L3, GP73, and DCP). These models can be used as a preliminary screening tool to distinguish early stage HCC patients from LC patients, thus improving prevention and treatment of HCC.

## RESULTS

### Serum levels of AFP, AFP-L3, GP73, and DCP as HCC diagnostic markers

347 HCC and LC patients were recruited and divided into three groups: 114 advanced HCC patients, 81 early stage HCC patients, and 152 LC patients. The demographic data of the patients are shown in Table 1. There were no significant differences in age, HBV infection rate, history of infection, and liver function indexes (bilirubin and alanine transaminase) among the three groups. However, there were significant differences in gender, serum albumin levels, and prothrombin time (p<0.05). The data indicated that male patients were at a higher risk to develop HCC, and LC patients had a worse liver synthesis function compared with HCC patients.

**Table 1:** The demographic data of the patients

	Advanced HCC	Early HCC	LC	P value
Male/ Female	97/17	65/16	101/51	0.001
Age (yr)	53(21-74)	56(36-81)	51(23-83)	0.154
HBV infection/others	90/14	69/12	138/14	0.377
History of infection (yr)	10.9±7.5	14.1±6.5	14.7±8.2	0.183
Albumin (g/L)	33.4±6.0	33.8±6.8	30.7±6.3	0.001
Bilirubin (μmol/L)	25.4(4.6-520.6)	20.8(6.0-141.0)	24.9(4.8-423.3)	0.209
Prothrombin time(s)	13.3(10.7-22.2)	13.1(10.6-21.9)	13.9(10.2-23.2)	0.001
Alanine transaminase(IU/L)	44(9-1194)	39(7-263)	42(10-1260)	0.120

**Figure 1:**
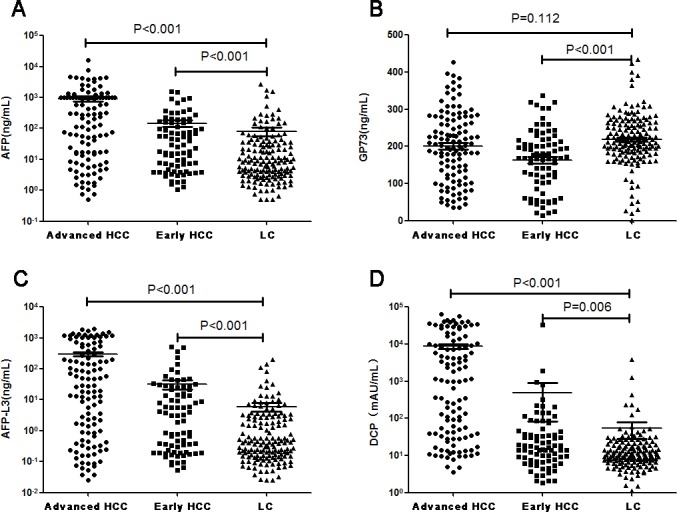
The difference in AFP (A), GP73 (B), AFP-L3 (C), and DCP (D) levels between advanced HCC, early stage HCC, and LC patients.

To evaluate the diagnostic value of the four serum tumor markers (AFP, AFP-L3, GP73, and DCP) in the progression of HCC, we measured their concentrations in serum of all patients. The serum levels of all four markers differed (p<0.05) between early stage HCC patients and LC patients. AFP, AFP-L3, and DCP also showed significant changes between advanced HCC patients and LC patients (p<0.05; Table 2). The serum levels of AFP, AFP-L3, and DCP gradually increased during the progression of cirrhosis to HCC. However, the serum GP73 levels in LC patients were higher than in advanced and early stages HCC patients; the early stage HCC patients had the lowest serum levels of GP73. In addition, there was no significant difference between advanced HCC patients and LC patients (p=0.112; Figure 1). In order to determine whether GP73 could be used in the diagnosis of HCC, we compared serum GP73 levels in LC patients and patients with all stages of HCC. The levels of GP73 differed (p<0.001, Z=-3.728) between all stages HCC patients and LC patients.

**Table 2:** The statistical analysis of the levels of serum markers between three groups

Markers	Advanced HCC Group	Early HCC Group	LC Group	Advanced HCC vs LC		Early HCC vs LC	
				Z Value	P Value	Z Value	P Value
AFP(ng/mL)	224 .0(0.5-16488.0)	29.5(1.0-1574.0)	8.4(0.5-2659.0)	-7.190	<0.001	-3.773	<0.001
GP73(ng/mL)	202.2 (35.4-427.4)	168.5 (13.1-337.5)	214.0(1.6-434.5)	-1.591	0.112	-5.103	<0.001
AFP-L3(ng/mL)	26.96(0.03-1981.00)	3.25(0.05-518.00)	0.42(0.03-204.4)	-7.566	<0.001	-4.330	<0.001
DCP(mAU/mL)	995.5(0.6-64091.0)	16.3(0.2-33210.0)	10.8(0.2-3778.4)	-10.426	<0.001	-2.736	0.006

### Serum levels of AFP, AFP-L3, GP73, and DCP as HCC diagnostic markers in cirrhotic patients

**Figure 2:**
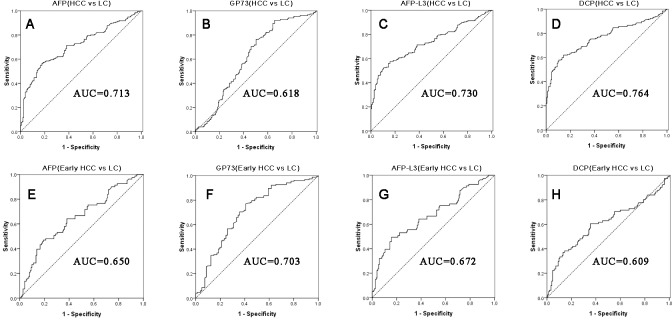
The ROC curves of AFP, GP73, AFP-L3, and DCP for diagnosis of all stages HCC and early stage HCC from the cirrhotic patients Figures **A-D** demonstrate AFP, GP73, AFP-L3 and DCP diagnostic performance in all stages HCC, while Figures **E-H** are in early stage HCC.

ROC analysis was used to determine whether the serum AFP, AFP-L3, GP73, and DCP levels are powerful to diagnose HCC in the cirrhotic population, as measured by the AUROC. The optimal cut-off values were determined with the maximum sum of sensitivity and specificity. As shown in Figures 2A-2D, which illustrate diagnostic performance of the four serum markers to differentiate all stages of HCC patients (including early and advanced HCC) from LC patients, DCP levels achieved a better diagnostic performance than the levels of the other three markers; AUROC for DCP was 0.764. However, for the diagnosis of early stage HCC, GP73 levels demonstrated a better performance (AUC=0.703; Figures 2E-2H), indicating that GP73 has a superior early diagnostic ability than the other markers. As expected, AFP, AFP-L3, and DCP had a relatively better diagnostic performance for HCC than for early stage HCC. Table 3 shows that AFP-L3 has the best specificity and GP73 has the best sensitivity in early stage HCC, as well as in all stages of HCC.

**Table 3:** The diagnostic performance of four markers and ANN models for all stages HCC and early stage HCC cases in cirrhotic patients

Markers	HCC vs LC					Early HCC vs LC				
	AUC	p Value	Cut-off	Sensitivity	Specificity	AUC	p Value	Cut-off	Sensitivity	Specificity
AFP(ng/mL)	0.713(0.659-0.767)	<0.001	49.4	0.564	0.822	0.650(0.575-0.725)	<0.001	43.7	0.469	0.809
GP73(ng/mL)	0.618(0.559-0.677)	<0.001	148.6	0.921	0.344	0.703(0.630-0.776)	<0.001	181.4	0.770	0.593
AFP-L3(ng/mL)	0.730(0.678-0.782)	<0.001	8.028	0.523	0.895	0.672(0.597-0.748)	<0.001	3.813	0.494	0.842
DCP(mAU/mL)	0.764(0.714-0.814)	<0.001	28.1	0.621	0.855	0.609(0.527-0.690)	0.006	13.7	0.605	0.645
MLP-Models	0.753(0.701-0.806)	<0.001	0.5	0.697	0.809	0.692(0.616-0.768)	<0.001	0.5	0.469	0.914
RBF-Models	0.742(0.688-0.795)	<0.001	0.5	0.733	0.750	0.659(0.582-0.735)	<0.001	0.5	0.481	0.836

### Development of neural network models to differentiate LC and HCC patients

**Figure 3:**
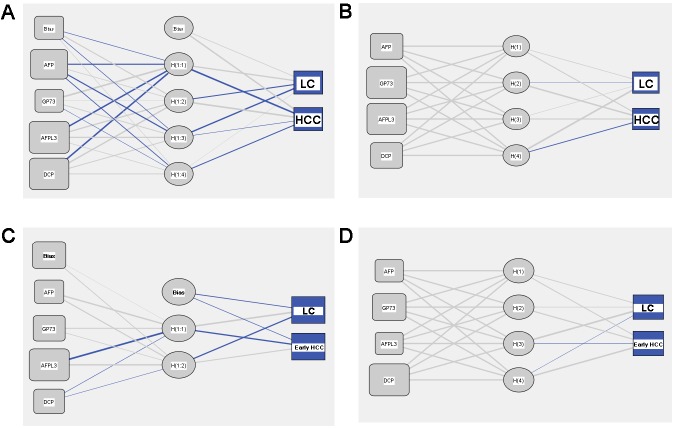
Architecture of neural network models **(A)** MLP model for differentiating all stages HCC from LC patients; **(B)** RBF model for differentiating all stages HCC from LC patients; **(C)** MLP model for differentiating early stage HCC from LC patients; **(D)** RBF model for differentiating early stage HCC from LC patients. The blue lines represent synaptic weight>1, the grey lines represent synaptic weight<1.

**Figure 4:**
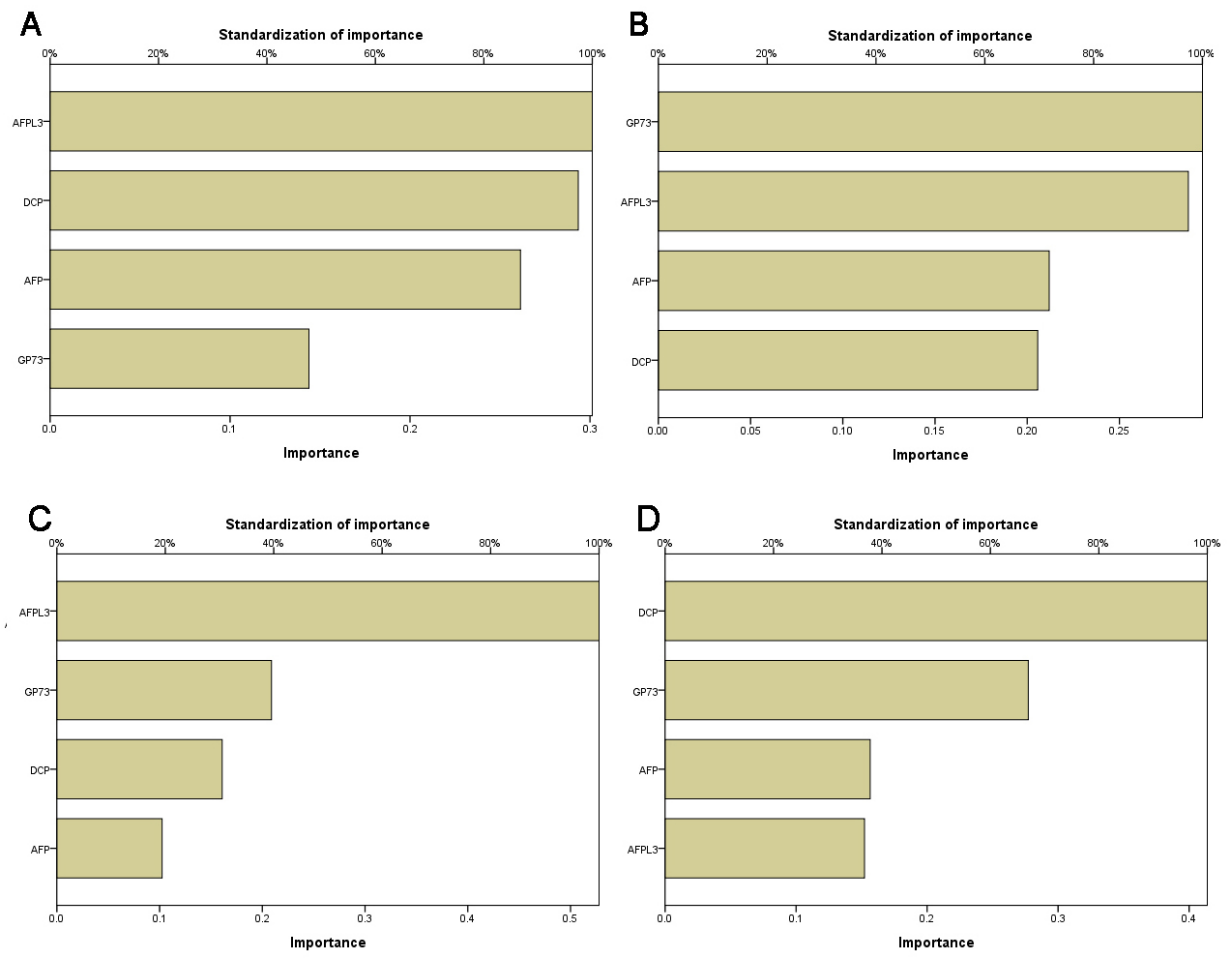
The relative importance of the four markers to the diagnostic models **(A)** MLP model importance histogram for LC vs all stages HCC; **(B)** RBF model importance histogram for LC vs all stages HCC; **(C)** MLP model importance histogram for LC vs early stage HCC; **(D)** RBF model importance histogram for LC vs early stage HCC.

**Figure 5:**
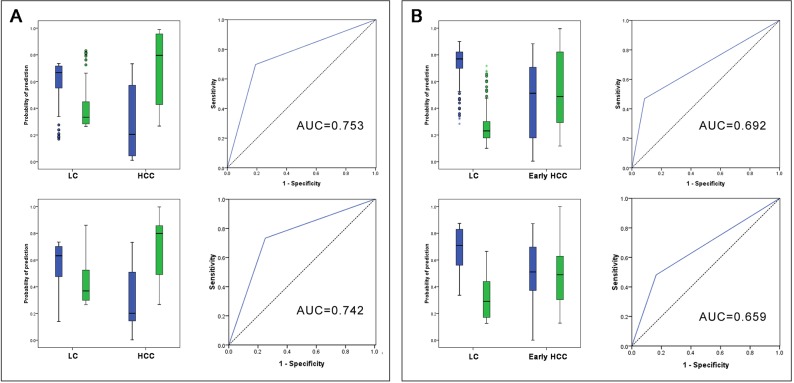
Prediction probability histograms and ROC curves for neural network models **(A)** LC vs all stages HCC; **(B)** LC vs early stage HCC. The upload, MLP models; the download, RBF models. Green columns represent diagnosed HCC samples; blue columns represent diagnosed LC samples.

We used multilayer perceptron (MLP) and radial basis function (RBF) neural networks to construct the diagnostic models. Figure 3A and 3B show the architecture of two models; both of them included four input layers neurons and two output layers neurons. For the MLP model, 74.6% training samples, 70.8% testing samples, and 82.1% holdout samples were correctly diagnosed (Table 4). Analysis of the importance of the four serum markers showed that AFP-L3 was the most important variable in MLP model (100 %); the following variables were DCP (97.4%), AFP (86.8%), and GP73 (47.7%) (Figure 4A). For the RBF model, 72.8% training samples, 81.0% testing samples, and 70.6% holdout samples were correctly diagnosed (Table 4). GP73 was the most important variable, followed by AFP-L3 (97.4%), AFP (71.8%), and DCP (69.7%) (Figure 4B). Prediction probability histograms (Figure 5A) showed that the MLP model could accurately recognize LC patients. In contrast, the RBF model had a better recognition ability for HCC patients. The AUROCs of MLP and RBF models were 0.753 and 0.742 in HCC diagnosis, respectively. These two models had a better diagnostic performance than the serum levels of AFP, AFP-L3, and GP73. Table 3 shows that the two models achieved a higher sensitivity than the individual serum biomarkers, even though their specificity was somewhat decreased.

**Table 4:** Diagnostic results of two models for all stages HCC and LC patients

Group	Diagnosis	MLP Model			RBF Model		
		LC	HCC	Accuracy	LC	HCC	Accuracy
Traning set	LC	82	17	82.8%	76	29	72.4%
	HCC	43	94	68.6%	39	106	73.1%
	Total percentage	53.0%	47.0%	74.6%	46.0%	54.0%	72.8%
Test set	LC	25	10	71.4%	23	6	79.3%
	HCC	11	26	70.3%	6	28	82.4%
	Total percentage	50.0%	50.0%	70.8%	46.0%	54.0%	81.0%
Holdout set	LC	16	2	88.9%	15	3	83.3%
	HCC	5	16	76.2%	7	9	56.3%
	Total percentage	53.8%	46.2%	82.1%	64.7%	35.3%	70.6%

### Development of neural network models to differentiate LC and early stage HCC patients

Since prediction or early HCC diagnosis is more important than prediction of late-stage diagnosis, the main purpose of this study was to establish sensitive and accurate early stage HCC diagnostic models. We used MLP and RBF neural networks to construct the diagnostic models, using the early stage HCC and LC as the two model output layers neurons. As shown in Table 5, the accuracies of the MLP model for training set, test set, and holdout set were 75.8%, 81.3%, and 66.7%, respectively. The order of importance was AFP-L3 (100%), GP73 (39.6%), DCP (30.5%), and AFP (19.4%) (Figure 4C). For the RBF model, the accuracies for the three sets were 71.1%, 70.0%, and 75.0%, respectively. The order of importance was DCP (100%), GP73 (67.0%), AFP (37.8%), and AFP-L3 (36.8%) (Figure 4D). Figure 5 illustrates that the two models could accurately identify LC patients, but could not identify early stage HCC patients. Their AUROCs were 0.692 and 0.659, respectively. However, in spite of the high specificities of both models, they had a lower sensitivity compared to the individual biomarkers (Table 3), indicating that they may increase the risk of missed HCC diagnosis.

**Table 5:** Diagnostic results of two models for early stage HCC and LC patients

Group	Diagnosis	MLP Model			RBF Model		
		LC	Early HCC	Accuracy	LC	Early HCC	Accuracy
Traning set	LC	98	6	94.2%	91	18	83.5%
	Early HCC	33	24	42.1%	32	32	50.0%
	Total percentage	81.4%	18.6%	75.8%	71.1%	28.9%	71.1%
Test set	LC	29	5	85.3%	23	5	82.1%
	Early HCC	4	10	71.4%	7	5	41.7%
	Total percentage	68.8%	31.3%	81.3%	75.0%	25.0%	70.0%
Holdout set	LC	12	2	85.7%	13	2	86.7%
	Early HCC	6	4	40.0%	3	2	40.0%
	Total percentage	75.0%	25.0%	66.7%	80.0%	20.0%	75.0%

## DISCUSSION

In recent years, many promising candidate biomarkers for HCC have been identified, but most of them have not been applied in the clinical diagnosis due to their limited practicability and high cost [25–29]. Currently, AFP and imaging technology, such as ultrasound or computed tomography, are the two methods mainly used to diagnose HCC in hospitals. AFP has been used as an HCC serum biomarker for many years, but its sensitivity is only about 40%-65% [30]. AFP-L3, which is the main glycoform of AFP in the serum of HCC patients, has been proven to be an excellent biomarker with sensitivity of 75% to 97%. High levels of AFP-L3 have been associated with poor differentiation, worse liver function, and larger tumor mass. Some studies have suggested that the AFP-L3/AFP ratio might be more helpful in diagnosis and prognosis of HCC than the AFP-L3 values [31, 32]. However, Miura and his coworkers have shown that the AFP-L3 levels cannot provide an entirely satisfactory solution to detect HCC at the early stage [33]. Our results show that the serum AFP-L3 levels gradually increase during the progression of cirrhosis to HCC. The AUROCs of AFP-L3 for HCC and early stage HCC were 0.730 and 0.672, respectively. The serum AFP-L3 levels in early stage HCC patients (median=3.25 ng/mL) were higher than in LC patients (median=0.42 ng/mL, p<0.05), suggesting that AFP-L3 may have a clinical value for the diagnosis of early HCC.

GP73 is a resident Golgi-specific membrane protein expressed by biliary epithelial cells in the liver. A meta-analysis study has suggested that GP73 is a valuable serum marker that seems to be superior to AFP and can be useful in the diagnosis and screening of HCC [34]. However, our results indicate that GP73 is elevated not only in HCC, but also in LC; the concentration of GP73 in HCC (median=202.2 ng/mL) was lower than in LC patients (median=214.0 ng/mL, p<0.05). The results of Tian et al. [35] are in agreement with our study; the median serum levels of GP73 were 107.3 μg/L in the HCC group and 141.2 μg/L in the LC group. Previous studies have shown that GP73 gene and protein levels gradually increase in chronic liver diseases; not only in hepatocytes, but also in activated hepatic stellate cells, which are the major cell type in liver cirrhosis [36–38]. Since we have observed maximal GP73 concentrations in liver cirrhosis rather than in HCC, these data suggest that GP73 might be regarded as a biomarker for liver cirrhosis rather than HCC. In addition, we have found that GP73 is the most sensitive biomarker to differentiate between early-stage HCC and LC patients; its sensitivity and AUROC are 0.770 and 0.703, respectively. However, the low specificity of GP73 limits its potential as an HCC biomarker, even though its sensitivity is extremely high.

DCP is an abnormal prothrombin molecule, which is induced by vitamin K absence II (PIVKAII), and may play an important role in promoting malignant HCC proliferation. Previous studies have shown that the serum DCP levels in patients with benign and malignant liver diseases are higher than in healthy people, suggesting that DCP might have a higher diagnostic sensitivity compared to AFP [39–42]. Our results show that the DCP levels in LC and all stages HCC patients have sensitivity of 62.1% and specificity of 85.5%. For early stage HCC, the sensitivity and specificity of DCP are 60.5% and 64.5%, respectively. DCP has been suggested as a biomarker for early stage HCC detection. Our results demonstrate that the DCP sensitivity is about 60% for HCC at an early stage. Although our study demonstrates that the sensitivity of DCP is higher than that of AFP in the diagnosis of early stage HCC, the DCP specificity is relatively low.

Artificial neural network (ANN) is ideal for diagnosis or prediction of disease in individuals, since it fits a nonlinear correlation between input and output variables [43–47]. There are various methods used for training of the network; MLP and RBF are the most common. Here, we have used these two networks to develop models for distinguishing early stage HCC from LC patients. Four serum biomarkers, AFP, AFP-L3, GP73, and DCP, have been used in the neural network modeling. The trained MLP and RBF models for recognition of all stages HCC and early stage HCC are presented in Tables 4 and 5. Single serum biomarkers are insufficiently precise for the diagnosis of HCC, but using their combinations greatly increases the accuracy. The HCC diagnostic models that we have developed have excellent diagnostic potential: their accuracy exceeded 80%, and their sensitivity was improved compared to single biomarkers. However, the early-stage HCC diagnostic models have a relatively low sensitivity, which may lead to some missed diagnoses. Combination of both models should be a more reliable approach for the diagnosis of early-stage HCC. In the clinical practice, the HCC RBF model (sensitivity=73.3%) might be used as a screening tool for detection of early stage HCC and its differentiation from LC, while the early stage MLP HCC model (specificity=91.4%) might be applied to exclude false positives. This strategy should not only improve HCC detection rates, but also reduce false positives in early HCC stages.

In conclusion, we have evaluated the potential of AFP, AFP-L3, GP73, and DCP serum biomarkers for HCC diagnosis, and developed diagnostic models using these biomarkers and MLP and RBF neural networks to differentiate HCC and early stage HCC from LC patients. These models can differentiate HCC and early stage HCC from liver cirrhosis. Future studies will be necessary to test their potential for clinical benefit in HCC patients.

## MATERIALS AND METHODS

### Human subjects

347 subjects were recruited from outpatients and inpatients of the 302 Military Hospital of China (114 advanced HCC patients, 81 early stage HCC patients, and 152 LC patients) from February 2013 to December 2015. The diagnosis of HCC was made by liver histopathology or MRI based on the guidelines from the ministry of health of the People's Republic of China [48]. The diagnosis of LC was based on clinical, laboratory and imaging evidence based on the guidelines from the Chinese Society of Hepatology and the Chinese Society of Infectious Diseases [49, 50]. The stage of tumor was based on BCLC staging system; patients with BCLC 0-A stages were denoted as the early stage HCC group, and those with BCLC B-D stages were denoted as the advanced HCC group [8]. Patients with the following conditions were excluded: Patients with other systemic diseases, such as diabetes or hypertension, and patients with severe complications, such as upper gastrointestinal bleeding or hepatic encephalopathy. The study procedures were approved by the ethics committee of the 302 military hospital of China and written informed consent was obtained from all subjects.

### Laboratory tests

The serum samples were collected in 5 mL vacuum blood collection tubes without anticoagulant, then centrifuged for 5 min at 12,000g at room temperature. Serum concentrations of AFP and GP73 were measured using chemiluminescent immunoassay kits (Hotgen Biotech Co, China). For AFP-L3, the serum was first fractionated on lectin-affinity column, and Lens culinaris agglutinin selective elution was used to assay AFP-L3 by chemiluminescent immunoassay. Serum DCP levels were measured by Architect i2000 immunoassay analyzer (ARCHTECT PIVKA-II, Abbott Co, USA). Clinical tests were performed by an AU5400 automatic biochemical analyzer (Beckman Co, USA).

### Development of the neural network models

Two types of ANN models, MLP and RBF, were developed by SPSS 17.0 Neural Network module. MLP and RBF are two popular architectures used in ANN; they are three-layer neural networks with input layer, hidden layer, and output layer. MLP is always trained by a back-propagation algorithm. When a neural group is provided with data through the input layer, the neurons in this first layer propagate the weighted data and randomly selected bias through the hidden layers. Once the net sum at a hidden node is determined, an output response is provided at the node using a transfer function. RBF neural network is a multilayer feed-forward network that can be used to identify nonlinear model effectively. The hidden layer transforms the data from the input space to the hidden space using a non-linear function. The output layer, which is linear, yields the response of the network [51].

In this study, four selected variables (AFP, GP73, AFP-L3, and DCP) were used as the input layer neurons, and two variables (LC and early stage HCC or all stages HCC) were used as the output layer neurons. All subjects were randomly divided into a training set, a test set and a holdout set at the ratio of 7:2:1. Training set is used to train the network, holdout set is used to assess model's performance, and test set is used to validate the results.

### Statistical analysis

All statistical analyses were performed using the software SPSS 17.0. To assess the role of four tumor markers as diagnostic markers for LC or HCC, receiver operating characteristic curves (ROC) were plotted, and the area under the curve (AUROC) was calculated. Data with normal distribution were analyzed with Student's t tests or one-way analysis of variance; other data were analyzed by the Wilcoxon or Kruskal-Wallis tests.
